# The Role of Crosslinker Content of Positively Charged NIPAM Nanogels on the In Vivo Toxicity in Zebrafish

**DOI:** 10.3390/pharmaceutics15071900

**Published:** 2023-07-07

**Authors:** Roberta Bilardo, Federico Traldi, Caroline H. Brennan, Marina Resmini

**Affiliations:** 1 Department of Chemistry, School of Physical and Chemical Sciences, Queen Mary University of London, London E1 4NS, UK; r.bilardo@qmul.ac.uk (R.B.); f.traldi@qmul.ac.uk (F.T.); 2 School of Biological and Behavioural Sciences, Queen Mary University of London, London E1 4NS, UK; c.h.brennan@qmul.ac.uk

**Keywords:** nanogels, zebrafish, toxicity, positive surface charge, NIPAM

## Abstract

Polymeric nanogels as drug delivery systems offer great advantages, such as high encapsulation capacity and easily tailored formulations; however, data on biocompatibility are still limited. We synthesized *N*-isopropylacrylamide nanogels, with crosslinker content between 5 and 20 mol%, functionalized with different positively charged co-monomers, and investigated the in vivo toxicity in zebrafish. Our results show that the chemical structure of the basic unit impacts the toxicity profile depending on the degree of ionization and hydrogen bonding capability. When the degree of crosslinking of the polymer was altered, from 5 mol% to 20 mol%, the distribution of the positively charged monomer 2-*tert*-butylaminoethyl methacrylate was significantly altered, leading to higher surface charges for the more rigid nanogels (20 mol% crosslinker), which resulted in >80% survival rate (48 h, up to 0.5 mg/mL), while the more flexible polymers (5 mol% crosslinker) led to 0% survival rate (48 h, up to 0.5 mg/mL). These data show the importance of tailoring both chemical composition and rigidity of the formulation to minimize toxicity and demonstrate that using surface charge data to guide the design of nanogels for drug delivery may be insufficient.

## 1. Introduction

The development of nanomaterials as drug delivery systems is increasingly attractive thanks to the advantages that these materials bring, such as high loading capacity [1,2,3,4], site-specific targeting [5,6], and controlled drug release via external stimuli, such as temperature and pH [7,8]. Covalently crosslinked acrylamide-based nanogels (NGs), with their highly swellable matrices, have high colloidal stability and higher biocompatibility compared to other nanomaterials [9,10,11,12,13,14,15,16]. In addition, the synthetic methods are simple, an important feature that allows the formulations to be easily tailored, facilitating not only the uploading of different drugs, but also the evaluation of how changes in formulation result in different properties [17,18,19,20].

The relationship between the formulation and properties of NGs has attracted considerable research, particularly given the importance of chemical structure and morphology in influencing the interaction with biological entities, in vivo, resulting in a protein/biomolecular corona [21]. We investigated the impact of changes in formulations and structure on the interfacial behavior of *N*-isopropylacrylamide (NIPAM) NGs, using a combination of neutron reflectivity and atomic force microscopy [22], and correlated formulations with changes in thermoresponsive properties [23], together with the role of monomer reactivities [24]. More recently, we reported our results on the role that charges play on biocorona formation with acrylamide-based NGs [25].

Despite advances in understanding the structure and properties of NGs, their use in clinical applications remains limited by the lack of comprehensive knowledge of their biocompatibility and long-term toxicity [26]. Important studies have highlighted how the toxicity of nanoparticles may be correlated to their smaller size (and higher surface-to-volume ratio) [27,28,29], chemical composition [30], and their surface hydrophobicity [31,32,33] and charge [34,35]. In particular, the surface charge of nanoparticles has been reported to play a key role in controlling their affinity for biological systems. Compared to neutral nanoparticles, positively charged counterparts generally show greater interaction with biomolecules, leading to uptake by the immune system [36,37] and stronger adsorption on biological membranes via electrostatic interactions with the negatively charged glycoproteins of the mammalian cell surface [38]. This has been associated with a higher cellular internalization for positively charged particles and, consequently, greater toxicity [39,40], as found for both inorganic [41,42,43,44] and organic nanoparticles [45,46]. Nevertheless, the interest in this type of nanomaterial is still high due to its higher cellular permeation efficiency and consequent high potential for efficient transport to tissues.

The biocompatibility of nanomaterials is dependent on several physico–chemical properties, and both in vitro and in vivo data are often necessary for the development of drug nanocarriers. Among the in vivo models currently preferred, the zebrafish is very convenient for the assessment of toxicity and safety, being an intermediate step between cell-based evaluation and conventional animal testing on mammals [47,48]. Introduced by Streisinger in the 1980s [49], this animal model is characterized by a similar toxicity profile to mammals; thus, in line with the general desire to reduce mammalian animal testing, the zebrafish embryo is emerging as a viable alternative to prioritize compounds for developmental toxicity testing in higher vertebrates. Moreover, it presents several advantages, such as small size, simple natural habitat, easily bred in large numbers, and low cost [50,51], which make the use of this animal model technically and economically convenient. The evaluation of new materials is facilitated by their absorption from the medium through their skin and gills, at the embryonic and early larval stage, and by their rapid development, with the formation of most of their organs and tissues by 5 days post fertilization (dpf) [51], which allows detection of toxic effects at an early developmental stage, using a relatively low number of animals, in agreement with the 3Rs principle.

In this work, we present our study on the role that crosslinker content and surface charge have on the toxicity of NIPAM-based NGs, evaluated in the zebrafish model up to 4 dpf. The impact of the addition of the positively charged monomer 2-*tert*-butylaminoethyl methacrylate to NIPAM formulations with varying degrees of *N*,*N*′-methylenebisacrylamide (MBA) as a crosslinker was studied by dynamic light scattering, electrophoretic light scattering, and UV-Vis spectroscopy. The changes in properties were then correlated with the in vivo toxicity studies of environmental exposure of zebrafish embryos starting from 2 to 2.5 h post fertilization. The role of charge was further investigated using two different functional monomers, bearing higher surface charge but with different chemical structure, studying the changes in properties and correlating them to in vivo toxicity to identify possible trends.

## 2. Materials and Methods

### 2.1. Materials

All chemicals were used as received unless otherwise stated. *N*,*N*′-methylenebisacrylamide (MBA), 2-(*tert*-butylamino)ethyl methacrylate (*t*-BAEMA, 97%, containing monomethyl ether hydroquinone MEHQ as inhibitor), 3-acrylamidopropyltrimethylammonium chloride (ATC, 75% *w*/*v* in water), and 1,2,4,5-tetramethylbenzene (TMB, 98%) were purchased from Sigma Aldrich (Gillingham, UK). *N*-isopropylacrylamide (NIPAM, 97%) and 2,2′-azobisisobutyronitrile (AIBN, 98%) were purchased from Sigma-Aldrich and recrystallized in n-hexane and methanol, respectively. *N*-(3-methacrylamidopropyl) guanidinium chloride (GUA) was synthesized as previously reported [25]. Anhydrous dimethyl sulfoxide (DMSO, 99.8%) was obtained from Acros Organics Chemicals (Geel, Belgium), while deuterated DMSO-d_6_ (>99% D, (CD_3_)_2_SO) used for the monomer conversion analysis by ^1^H NMR was purchased from Goss Scientific (Crewe, UK). Regenerated cellulose dialysis membrane (MWCO 3.5 kDa, width 34 mm, diameter 22 mm) was purchased from Medicell Membranes Ltd. (London, UK). Deionized water, used for both NGs purification and characterization, was obtained using the Elga Option PURELAB Water Purification System. Fish water is a saltwater solution comprised of sodium bicarbonate, marine salts and calcium sulfate in a ratio of 7.5:1.8:0.84 g in 100 L of reverse osmosis (R/O) purified water, and this was used as taken from the zebrafish maintenance water system. Polytetrafluoroethylene (PTFE) syringe filters with pore size 0.2 μm were obtained from Fisher Scientific (Loughborough, UK).

### 2.2. Synthesis of NGs

NGs were prepared by high dilution radical polymerization (HDRP) according to a previously reported procedure [24]. Monomers were solubilized, together with the crosslinker MBA, in anhydrous DMSO in a 10 mL Wheaton™ bottle. The volume of dry DMSO was adjusted to obtain the desired total monomer concentration C_M_ 1–3% (*w*/*w*) depending on the formulation. The initiator AIBN was then added to the polymerization mixture in concentrations of 1 or 2 mol% of total moles of double bonds present in the mixture.

The Wheaton™ bottle was sealed and the pre-polymerization mixture purged with N_2_. The solution was then placed in an oven at a temperature equal to 70 °C. After 24 h, the transparent NG solution was dialyzed against deionized water for 3 days with frequent changes (3 times per day) before being transferred to a 50 mL falcon tube and frozen in liquid N_2_. The frozen NG solution was lyophilized using an LTE Scientific Lyotrap freeze-drier (Greenfield, UK) to yield a fluffy white powder. All the NGs synthesized were stored in glass vials at room temperature.

### 2.3. Study of Monomer Conversions by ^1^H NMR

The quantification of the monomer conversions of the NGs was carried out by ^1^H NMR (Bruker AVIII 400 MHz BBO Probe), simultaneously to the NG synthesis, following a previously reported procedure [24]. Aliquots of 250 μL were drawn from the Wheaton™ bottle before (pre-polymerization, t = 0 h) and after (post-polymerization, t = 24 h) the 24 h polymerization and mixed with 250 μL of DMSO-d_6_ in an NMR tube. The internal standard TMB, solubilized in DMSO-d_6_ (concentration between 0.15 and 0.19 M), was added to both NMR tubes to obtain a molar concentration ratio equal to 1:4 with NIPAM. The ^1^H NMR spectra were acquired using a method suppressing the non-deuterated solvent signal (δ_DMSO_ = 2.50 ppm) and were then phased and integrated manually with the Bruker TopSpin 4.0.6 software. To calculate the concentration of unreacted monomers and MBA in the initial and final polymerization solutions, the intensities of monomer peaks (5.55 ppm for NIPAM, 5.67 ppm for *t*-BAEMA, 5.66 ppm for GUA, and 5.63 ppm for MBA) were compared to the intensity of TMB peak at 6.88 ppm.

### 2.4. Nanoparticle Size by Dynamic Light Scattering

The hydrodynamic diameter (Dh) of NGs was measured by DLS at 25 °C using a Zetasizer Nano Ultra operated with the ZS Xplorer software (version 1.5.0.163) (Malvern Instruments Ltd., Malvern, UK). NGs colloidal solutions (concentration 1 mg/mL) were prepared by dissolving the dry NG powder in either deionized water or fish water, followed by sonication for 10 min at room temperature, and filtration through 0.2 μm PTFE syringe filters. To avoid any contamination due to the filtration, the filters were preconditioned with 1 mL of the solvent and the first 3 drops of filtered NG solution discarded. All the DLS measurements were carried out in triplicate using backscatter angle mode (174.7°), allowing at least 4 min for the sample temperature to equilibrate prior to each measurement.

To study the colloidal stability of the nanogels, after obtaining nanogel solutions in fish water (pH = 7) at 0.5 mg/mL in the same way as described for hydrodynamic diameter characterization, samples were transferred into a DLS cuvette and incubated at 28 °C in a water bath for up to 96 h. Then, triplicate DLS measurements (number distribution) were collected at 0, 24, and 96 h without removing the sample from its cuvette; the colloidal stability of the nanogels was evaluated by comparing the size of the nanogels at different times compared to their initial size (0 h).

### 2.5. Nanoparticle Surface Charge by Electrophoretic Light Scattering

The electrophoretic mobility (μ) of positively charged NGs was investigated by ELS at 28.5 °C, using a Zetasizer Nano Ultra operated with the ZS Xplorer software (version 1.5.0.163) (Malvern Instruments Ltd., Malvern, UK). All the NGs were analyzed after dispersion in fish water (pH = 7) at a concentration equal to 1 mg/mL, sonication for 10 min, and filtration through 0.2 μm PTFE syringe filters. The samples were then transferred into disposable folded capillary cells (1080, Malvern Instruments Ltd., Malvern, UK) and the measurements performed after at least 4 min equilibration.

### 2.6. Thermoresponsive Properties of NGs by UV-Visible Spectroscopy

The volume phase transition temperatures (VPTTs) of the NGs were determined by turbidimetry assay, i.e., by measuring the light transmittance (at 500 nm) of the NG solutions as a function of temperature. The NG solutions, with concentration equal to 1 mg/mL, were prepared by dissolution of the dry NG in either deionized water or fish water followed by 10 min sonication and filtration with 0.2 μm PTFE syringe filters. The measurements were carried out in a temperature range between 25 and 65 °C, at a rate of 0.5 °C/minute, using a Cary 100 UV-Visible Spectrophotometer (Agilent Technologies, Santa Clara, CA, USA) coupled with a temperature controller. The raw data were analyzed using Origin2018 software, which allowed the plotting and fitting of the transmittance-temperature data with the sigmoidal Boltzmann fitting. For each measurement, the VPTT was considered equal to the temperature at which the curve presented its inflection point, after verifying the accuracy of this fitting in terms of adjusted R^2^ (≥0.99).

### 2.7. Zebrafish Husbandry and Maintenance

In the animal facility, wild type Tübingen (TU) strain zebrafish were maintained at 28.5 °C in a recirculating system (Tecniplast, UK) on a 14 h:10 h light/dark cycle. Fish were fed twice daily, with ZM-400 fry food (Zebrafish Management Ltd., Winchester, UK) in the morning and brine shrimp in the afternoon, following standard husbandry protocols [52]. For breeding, fish were placed in groups of 3 (2 females and 1 male) in sloping breeding tanks (Tecniplast, UK) overnight with a clear divider separating males from females. The divider was removed on the following morning to allow fish to spawn, and the eggs were collected between 2 and 2.5 h post fertilization (hpf), when they were sorted fertile from infertile and placed individually in the wells of 48-well plates (1 mL of fish water or NG solution in each well). The embryos/larvae were then incubated at 28.5 °C for the entire duration of the experiment. All procedures were performed in accordance with UK Animals (Scientific Procedures) Act. No regulated procedures were undertaken (as no experiments were continued past 4 dpf).

### 2.8. Evaluation of the Biocompatibility of NGs in Zebrafish

The effects of NGs on zebrafish were assessed by monitoring the morphology and development of the embryos/larvae immersed in NG solutions. NG solutions in fish water, having concentration between 0.01 and 0.5 mg/mL, were obtained by dilution from 1 mg/mL stock solutions, prepared following the same protocol described for the characterization studies. After collection and sorting, the embryos at 64–256 cell stage (2 to 2.5 hpf) were placed individually in the wells of a 48-well plate, immersed in 1 mL of NG solution or, in case of control, of fresh fish water, and housed in an incubator at 28.5 °C. Their morphology and development were monitored under a Leica M165 FC stereo microscope (Leica Microsystems, Germany) at 4, 24, 48, 72, and 96 h after the immersion and compared with the development of unexposed fish used as control. Each condition was tested on a group of 8 fish and repeated 3 times, when no damage due to the nanoparticle exposure was observed.

## 3. Results and Discussion

### 3.1. Synthesis and Characterization of Neutral and Positively Charged NGs

**Scheme 1:**
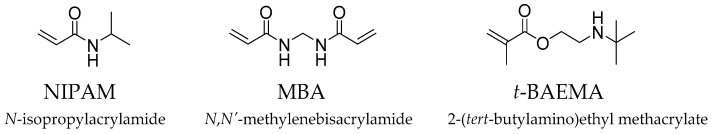
Chemical structure of monomers employed in the synthesis of NGs.

A small library of covalently crosslinked NGs was synthesized via high dilution radical polymerization (HDRP), which allows nano-sized gel particles to be obtained with tunable size without addition of surfactants [53]. To introduce a thermoresponsive behavior in the NGs, *N*-isopropylacrylamide (NIPAM), see Scheme 1, was employed as backbone monomer. NIPAM has been commonly employed to obtain thermoresponsive functional nanomaterials due to its lower critical solubility temperature (LCST) of 32 °C, which is close to body temperature. *N*,*N*′-methylenebisacrylamide (MBA) was used as the crosslinker in concentrations of 5–20 mol% to vary the flexibility of the particle and study its role on the physico–chemical and toxicological properties of the nanoparticles. All the formulations were prepared using 2,2′-azobisisobutyronitrile (AIBN) as the initiator, initially in concentration of 1 mol% (of the total moles of double bonds in the mixture) in DMSO. The particles were then purified via extensive dialysis, which ensures that the organic solvent is removed.

Positively charged NGs were obtained by introducing 10 mol% of 2-(*tert*-butylamino)-ethyl-methacrylate (*t*-BAEMA), which presents a weak basic character (pKa of 8 once polymerized [54]) that may reduce its impact on the thermoresponsive behavior of the NGs and limit toxicity. Good monomer conversions (71–88%) and chemical yields >70% (Table 1) were obtained for neutral NGs when a total monomer concentration (C_M_) of 1% (*w*/*w*) was employed, as found in previous works [24,55].

A lower yield compared to the overall monomer conversion is usually observed because of the loss of low-molecular-weight polymer particles during the purification step by dialysis (molecular cut-off 3.5 kDa). The addition of *t*-BAEMA in the formulation was associated with lower monomer conversions and yields at C_M_ 1 and 2% (Table S1), possibly due to a quenching effect of the methacrylate monomer. Increasing the C_M_ to the optimized value of 3% was found to bring both the overall monomer conversions and yields to satisfactory values, encouraging further characterization of the nanoparticles.

The hydrodynamic diameter (D_h_) of NGs is commonly determined by dynamic light scattering (DLS), which provides sizes based on the intensity of the scattered light; however, when analyzing particles with very small sizes, the presence of even very small amounts of larger particles, including dust, results in an over-estimation of their relative abundance in the sample. For this reason, in this case, size distribution by number provides a more realistic estimate of the particles’ diameters. The neutral NGs were found to be <10 nm in deionized water (Figure S1), consistent with previously reported NGs [25,55,56]; for MBA concentrations of 5, 10, and 20 mol%, a D_h_ of 3.6, 4.7, and 8.0 nm, respectively, was observed, showing the impact of the crosslinker on the size of the nanoparticles. A similar trend was seen for the NGs incorporating 10 mol% *t*-BAEMA, although the size of the particles was found to be overall slightly smaller (Table 1, Figure S2); this might be due to the hydrophobicity of the *t*-butyl substituent of the *t*-BAEMA unit, which is only partially ionized in water due to its weakly basic character. Given that the size of nanoparticles is affected by the nature of the media [56], the characterization was carried out in fish water, which is the medium used for the in vivo studies with zebrafish [52,57]. Data show that the size of the neutral NGs was mostly unaffected by the change in medium (Figure S3); however, positively charged NGs were found to have slightly larger D_h_ (Figure S4), possibly due to the lower pH of the fish water (around 7) compared to the unbuffered deionized water, leading to a larger degree of ionization of the *t*-BAEMA unit and, consequently, a more swollen NG matrix.

**Figure 1:**
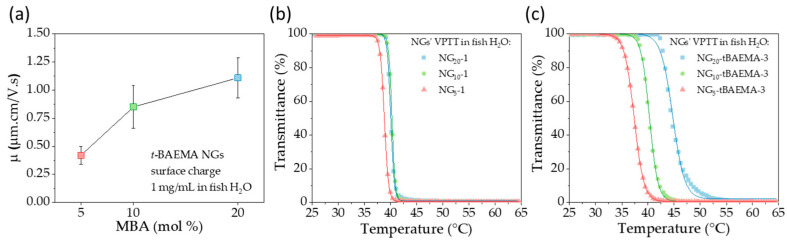
Characterization of NGs: (**a**) electrophoretic mobility analysis of NGs incorporating 10 mol% *t*-BAEMA in fish water (1 mg/mL). VPTT quantification of (**b**) neutral NGs, and (**c**) NGs incorporating 10 mol% *t*-BAEMA in fish water (1 mg/mL).

The electrophoretic mobility (μ) of the NGs was then measured to confirm the presence of the charge on the surface of the particles. Data in Figure 1a show that the NGs displayed a positive charge in fish water, which confirmed the presence of the *t*-BAEMA residues on the surface of the particle. Importantly, NGs with 5 mol% MBA displayed significantly lower μ values compared to those with 10 and 20 mol%, although no statistical difference between the 10 and 20% crosslinked NGs could be observed. These data suggest that the presence of a more loosely crosslinked matrix leads to a more uniform distribution of the *t*-BAEMA unit in the NG compared to the more surface-localized in the more crosslinked particles, thus leading to a lower surface charge.

To further investigate this finding, we characterized the thermoresponsive properties of the NGs by measuring their volume phase transition temperature (VPTT). The transmittance of NGs solutions (1 mg/mL) was monitored as a function of temperature in the range 25–65 °C using UV-Vis spectroscopy. The VPTT coincides with the temperature at which the transmittance of the sample drops to 50%. Neutral NGs with a different degree of crosslinking displayed similar VPTT values in fish water between 39 and 41 °C, while the VPTT of the *t*-BAEMA-NGs crosslinked with 5, 10, or 20 molar% of MBA were found to be 37.4, 40.3, and 44.7 °C, respectively, in fish water; these values are higher than the neutral NGs due to the presence of the positive charge, which increases the solvation of the NG’s polymer chains. Interestingly, a clear effect of the crosslinker content was observed in the case of the positively charged NGs, with higher VPTT values being observed for more crosslinked matrices. These data provide clear evidence that the more crosslinked NGs present better colloidal stability compared to the more loosely crosslinked NGs, possibly due to the larger content of positively charged units on their surface of the former, in agreement with the electrophoretic mobility data.

Having obtained the characterization data, the next step focused on evaluating whether the change in composition of the NGs and the difference in surface charge could influence the biocompatibility profiles of the particles. This was evaluated using zebrafish as an in vivo model.

### 3.2. Impact of Surface Chemistry and Morphology of NGs on their In Vivo Toxicity on Zebrafish Model

**Figure 2:**
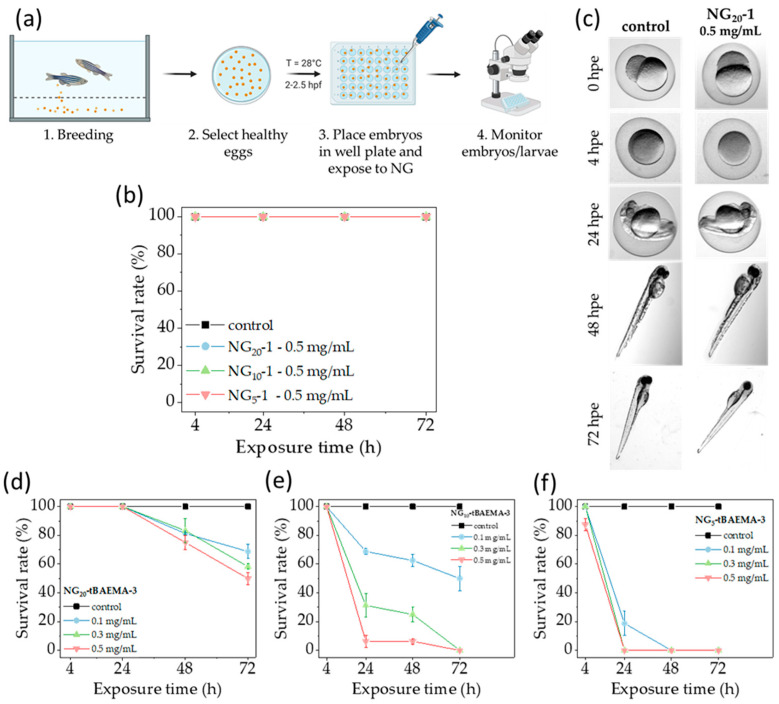
Toxicity studies of NGs on zebrafish larvae. (**a**) Procedure used to conduct in vivo toxicity tests. Neutral NGs showed (**b**) no toxicity on zebrafish, with (**c**) no effect on the development of the animals up to 72 h post exposure to the particles. Positively charged NGs showed increasing toxicity when the crosslinker content decreased from (**d**) 20 mol% to (**e**) 10 mol% to (**f**) 5 mol%. Survival rate data are reported as the average ± standard deviation of three independent experiments, and each condition was tested on eight embryos.

The in vivo toxicity of NGs was tested by direct environmental exposure on zebrafish up to 4 dpf, which involved adding the NGs to the medium where the zebrafish embryos were immersed (Figure 2a). The colloidal stability of the NGs in fish water was confirmed at the highest concentration studied (0.5 mg/mL) up to 96 hrs by DLS (Figure S5a–f). To evaluate the toxicity, the embryos/larvae were monitored under a stereomicroscope to check for any morphological deformities and/or any delay in their development. Moreover, after the hatching, the capability of swimming and response to touch stimuli were used as further parameters for healthy status. When the neutral NGs were tested at concentrations of 0.5 mg/mL, a 100% survival rate was observed for exposure times up to 72 h (Figure 2b) with no effects on the development of the embryos (Figure 2c for NG_20_-1, and Figure S6 for NG_10_-1 and NG_5_-1), suggesting that these formulations are well tolerated by the zebrafish model (regardless of their crosslinker content).

When the zebrafish embryos were exposed to positively charged NG_20_-tBAEMA-3 (20 mol% crosslinker) in concentrations ranging between 0.1 and 0.5 mg/mL, survival rates around 80% were recorded after only 48 h of incubation (Figure 2d), with higher concentration of 0.5 mg/mL leading to a drop to 50% survival rate at 72 h. This indicates that, as expected, the incorporation of a positively charged co-monomer in the NGs induces toxicity in the animal, likely a consequence of the interaction between the particle and the negatively charged cellular structures, resulting in a loss of cellular function and structure.

It is important to highlight that as the embryos are transparent, evidence of gross morphological changes can be visible generally without the need for staining. An example of images showing embryos exposed to toxic nanogels is shown in Figure S7.

Interestingly, positively charged NGs that presented a lower degree of crosslinker displayed a higher toxicity on the embryos (Figure 2e,f), with NG_5_-tBAEMA-3 leading to a 20% survival rate in 24 h even at the lowest concentration of 0.1 mg/mL. This result is somewhat counterintuitive, as characterization of the NGs indicated that NG_5_-tBAEMA-3 presented the lowest surface charge. However, NGs with less crosslinked matrices have been shown to possess higher flexibility and adaptability to interfaces [22,55]. The crosslinker, once incorporated in the matrix, does not impact toxicity on its own, as previously reported [16]. This, together with the data presented here, suggests that NGs with higher flexibility may be able to interact better with cellular structures, thus increasing the formulation’s toxicity. This finding also suggests that the characterization of nanoparticles may lead to inappropriate assumptions of their behavior in vivo, and that a complete characterization of NGs should be carried out before hypothesizing about their potential interactions with biological entities.

To further understand the role of the NG’s composition on their toxicity, we then evaluated how the addition of positively charged co-monomers having different structures affected the survival rate of the zebrafish embryo.

### 3.3. Impact of Positively Charged Group Structure on the Toxicity of NGs

**Figure 3:**
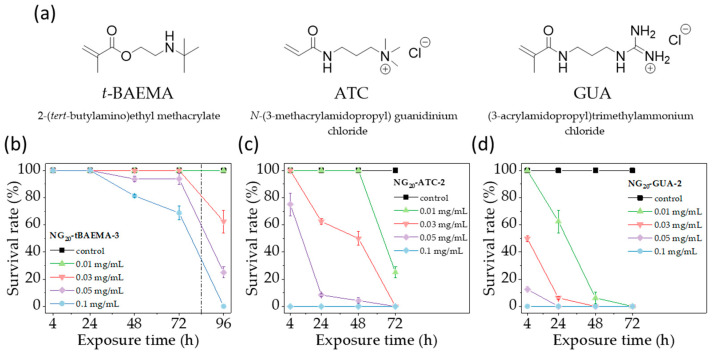
(**a**) Chemical structure of positively charged monomers used in the synthesis of NGs. (**b**) Toxicity study on positively charged NGs (20 mol% crosslinker) incorporating either (**b**) *t*-BAEMA, (**c**) ATC, or (**d**) GUA. Survival rate data are reported as the average ± standard deviation of three independent experiments, and each condition was tested on eight embryos.

Two additional positively charged monomers, 3-acrylamidopropyl-trimethylammonium chloride (ATC) and *N*-(3-methacrylamidopropyl)-guanidinium chloride (GUA), were separately added at concentrations of 10 mol% to the NGs formulations (Figure 3a). Given the lower toxicity observed for the more rigid *t*-BAEMA-based NGs, optimized formulations NG_20_-ATC-2 and NG_20_-GUA-2 were obtained using a fixed amount of 20 mol% of MBA (Table S2). Monomer conversions were found to be consistent with formulations containing *t*-BAEMA, although slightly lower chemical yields were observed for both NG_20_-ATC-2 (60%) and NG_20_-GUA-2 (54%).

The nanoparticles showed D_h_ around 7.5 nm (number distribution) in fish water (colloidally stable up to 96 h, Figure S5), which is comparable to NG_20_-tBAEMA-3. However, when electrophoretic mobility of the nanoparticles was analyzed (in fish water) values of 2 μm cm/V s were observed for both the ATC and GUA NGs, almost twice as high compared to the related *t*-BAEMA containing NG.

This result is likely due to the fully ionized nature of the ATC and GUA monomers in fish water, which leads to a higher amount of charged groups present on the surface of the NGs (despite all incorporating 10 mol% of charged co-monomer). Solutions of both NG_20_-ATC-2 and NG_20_-GUA-2 in fish water did not show any change in transmittance as a function of temperature up to 65 °C (Figure S8), a finding that further supports the observation that these NGs present a more charged and hydrated shell. These data indicate that the NGs incorporating three different types of positively charged groups are comparable in terms of size and display different surface properties that can be linked to their formulation. On this basis, we decided to evaluate these polymers in vivo.

We initially conducted toxicological studies on the 20 mol% crosslinked NG_20_-tBAEMA-3 at concentrations < 0.1 mg/mL; this was done to identify a suitable concentration at which the acute toxicity of the positively charged formulation could be considered negligible within the experimental time window. As observed in Figure 3b, NG_20_-tBAEMA-3 did not impact the survival rate of the zebrafish embryo (over 96 h) when used at a concentration of 0.01 mg/mL (Figure S9), indicating that the formulation could be used to compare the toxicity of the other two positively charged formulations.

The data in Figure 3 show that both NG_20_-ATC-2 and NG_20_-GUA-2 showed significant toxicity at 0.01 mg/mL; NG_20_-ATC-2 initially showed no toxicity for 48 h, but led to 20% survival rate after 72 h, demonstrating its more toxic profile compared to the *t*-BAEMA NG. NG_20_-GUA-2, on the other hand, immediately led to a loss of 40% of the initial embryos within 24 h, to then result in 0% survival rate after 72 h of exposure.

These results show that the NG toxicity depends on the type of positively charged monomer incorporated into the NG structure, with increasing toxicity being observed in the order *t*-BAEMA < ATC < GUA. NG characterization suggests that NG_20_-tBAEMA-3 has a lower surface charge compared to the other two NGs, likely due to its weakly basic character and consequent partial ionization, while, both NG_20_-ATC-2 and NG_20_-GUA-2 are fully ionized in the fish water medium, with similar surface charge. This indicates that their different toxicological profile might be determined by the chemical structure of the positively charged moieties. Indeed, GUA has been reported to strongly bind to the carboxylate groups of proteins [58] thanks to its ability to donate 2 hydrogen bonds; this is not possible in the case of the quaternary ammonium group in ATC, which may lead these NGs to form weaker bonds with proteins present on the surface of the cells. These data further expand the understanding of how the surface chemistry of nanoparticles requires careful design.

## 4. Conclusions

We synthesized and characterized covalently crosslinked NIPAM-based NGs, neutral or incorporating three different positively charged co-monomers, *t*-BAEMA, ATC, and GUA, with varying content of MBA (5–20 mol%). The NGs were obtained with a similar hydrodynamic diameter and polydispersity, while the surface charge and thermoresponsive behavior, resulting from the use of NIPAM as backbone monomer, was found to be consistent with their formulations. The data demonstrated that the crosslinker content directly influenced the location of the positively charged monomer *t*-BAEMA in the matrix, with higher values of surface charge observed when the crosslinker was increased from 5 mol% to 10–20 mol%.

The toxicity of the nanoparticles was evaluated by environmental exposure of the zebrafish larvae (up to 4 days post fertilization) to the different nanoparticles, and by monitoring the survival rate up to 96 h. Neutral NGs did not impact the survival of the zebrafish larvae, regardless of their rigidity, while the presence of the positively charged *t*-BAEMA in the matrix highlighted the key role played by the crosslinker content in influencing the toxicity. The nanogels with 20 mol% crosslinker, previously shown to have higher surface charge, resulted in >80% survival rate over a period of 48 h with concentrations up to 0.5 mg/mL, while the more flexible polymers with 5 mol% crosslinker were found to be highly toxic at the same concentrations (0% survival rate at 48 h).

The choice of the positively charged moiety also played an important role, where formulations having similar surface charge values but incorporating a quaternary ammonium group (ATC) led to lower toxicity compared to NGs incorporating the guanidinium group (GUA) as a consequence of different hydrogen bonding capabilities.

These results provide important insights into the roles that crosslinker content and functional groups’ structure play in influencing the toxicity of nanoparticles, as a result of influencing the position of charged units in the matrix, as well as determining its ability to interact with biomolecules, ultimately influencing their behavior in vivo. The development of new drug delivery systems based on nanoparticles requires a comprehensive characterization of nanoparticles that may lead to incorrect hypotheses regarding their behavior in vivo. The data revealed the importance of the degree of crosslinking, both in terms of its impact on the positioning of positively charged co-monomers in the matrix, and on the toxicity of the particle. The present study thus provides a tool to develop positively charged NGs with higher biocompatibility.

## Figures and Tables

**Table 1:** Chemical composition, monomer conversions (^1^H-NMR), and chemical yields of neutral and positively charged NGs based of NIPAM, with MBA in relative concentrations ranging between 5 and 20 mol%. For all the formulations, the initiator (AIBN) was 1% of the total moles of double bonds in the mixture *.

Nanogel	Composition (mol%)			C_M_ (%)	Monomer Conversion (%)				Yield (%)	D_h_ (nm)	
	NIPAM	MBA	*t*-BAEMA		NIPAM	MBA	*t*-BAEMA	Overall		D.I Water	Fish Water
NG_20_-1	80	20	-	1	86	97	-	88	81	8.0 ± 0.4	8.4 ± 0.6
NG_10_-1	90	10	-	1	82	93	-	83	74	4.7 ± 0.4	4.2 ± 0.5
NG_5_-1	95	5	-	1	70	86	-	71	71	3.6 ± 0.4	3.3 ± 0.5
NG_20_-tBAEMA-3	70	20	10	3	90	98	99	92	77	5.3 ± 0.3	8.5 ± 0.9
NG_10_-tBAEMA-3	80	10	10	3	87	95	99	89	66	3.9 ± 0.2	4.8 ± 0.8
NG_5_-tBAEMA-3	85	5	10	3	82	88	99	84	62	2.7 ± 0.3	4.3 ± 0.3

* The total monomer concentration (CM) was optimized to obtain good monomer conversions and yields. Hydrodynamic diameter (D_h_) by number distribution was obtained by DLS measurements on NG suspensions at 1 mg/mL in either deionized water or fish water at 25 °C.

## Data Availability

All data generated or analyzed during this study are included in this published article and its Supplementary Information.

## Supplementary Materials

The following supporting information can be downloaded at: https://www.mdpi.com/article/10.3390/pharmaceutics15071900/s1, Table S1. Chemical composition, monomer conversions (^1^H NMR), and chemical yields of positively charged NIPAM-based nanogels, containing 10 mol% *t*-BAEMA as functional monomer, before the optimization of the polymerization conditions. Figure S1. Triplicate DLS measurements of neutral nanogels with crosslinker content between 5 and 20 mol%: (a) NG_20_-1; (b) NG_10_-1; and (c) NG_5_-1 in deionized water (1 mg/mL, 25 °C) by intensity (blue), volume (green), and number (orange) distributions. Figure S2. Triplicate DLS measurements of *t*-BAEMA nanogels with crosslinker content between 5 and 20 mol%: (a) NG_20_-tBAEMA-3; (b) NG_10_-tBAEMA-3; and (c) NG_5_- tBAEMA-3 in deionized water (1 mg/mL, 25 °C) by intensity (blue), volume (green), and number (orange) distributions. Figure S3. Triplicate DLS measurements of neutral nanogels with crosslinker content between 5 and 20 mol%: (a) NG_20_-1; (b) NG_10_-1; and (c) NG_5_-1 in fish water with pH = 7 (1 mg/mL, 25 °C) by intensity (blue), volume (green), and number (orange) distributions. Inserts show correlogram of each triplicate measurement. Figure S4. Triplicate DLS measurements of *t*-BAEMA nanogels with crosslinker content between 5 and 20 mol%: (a) NG_20_-tBAEMA-3; (b) NG_10_-tBAEMA-3; and (c) NG_5_-tBAEMA-3 in fish water with pH = 7 (1 mg/mL, 25 °C) by intensity (blue), volume (green), and number (orange) distributions. Inserts show correlogram of each triplicate measurement. Figure S5. Colloidal stability studies of nanogels in fish water (pH = 7) at 28 °C (0.5 mg/mL). Triplicate DLS measurements (number distribution) of (a) NG_20_-1; (b) NG_10_-1; and (c) NG_5_-1; (d) NG_20_-tBAEMA-3; (e) NG_10_-tBAEMA-3; and (f) NG_5_-tBAEMA-3; (g) NG_20_-GUA-2; (h) NG_10_-ATC-2 were obtained after 0 (black), 24 (red), and 96 (blue) hours after reconstitution of the dry nanogel powders. Data show that nanogels are colloidal stable within the time frame of the in vivo toxicity studies. Figure S6. Stereomicroscopy assessment of the biocompatibility of neutral nanogels NG_10_-1 (10 mol% crosslinker, first row) and NG_5_-1 (5 mol% crosslinker, second row) in zebrafish up to 72 h post exposure (hpe). The morphology and development of the embryos/larvae, exposed by immersion in nanogel solutions in fish water (concentration 0.5 mg/mL), was monitored at 0, 4, 24, 48, and 72 h after the administration and compared to non-exposed embryos/larvae used as negative control. Table S2. Chemical composition, monomer conversions (^1^H NMR), and chemical yields of positively charged NIPAM-based nanogels containing 10 mol% of either *t*-BAEMA, or GUA, or ATC as functional monomer. All nanogels were synthesized keeping the crosslinker (MBA) content equal to 20 mol%. Figure S7. Examples of embryos/larvae dead after immersion into nanogels solutions for different times. Figure S8. UV-visible spectroscopy study of the thermoresponsive properties of the positively charged nanogels containing 20 mol% of crosslinker: (a) NG_20_-ATC-2 and (b) NG_20_-GUA-2 in fish water (concentration 1 mg/mL) in a temperature range between 25 and 65 °C. Figure S9. Stereomicroscopy assessment of the biocompatibility of the positively charged nanogel NG_20_-tBAEMA-3 (20 mol% crosslinker and 10 mol% of *t*-BAEMA), in zebrafish up to 96 h post exposure (hpe). The morphology and development of the embryos/larvae, exposed by immersion in nanogel solutions in fish water (concentration 0.01 mg/mL), was monitored at 0, 4 24, 48, 72, and 96 h after the administration and compared to non-exposed embryos/larvae used as negative control.
