# Supplementary material for: The Role of Crosslinker Content of Positively Charged NIPAM Nanogels on the In Vivo Toxicity in Zebrafish
Source: Pharmaceutics. 2023 Jul 7;15(7):1900. doi: 10.3390/pharmaceutics15071900 (PMC10383542; doi:10.3390/pharmaceutics15071900)
Supplement: Supplementary file 1 [file pharmaceutics-15-01900-s001.zip › pharmaceutics-2452643-supplementary.pdf]

Supplementary Materials

The Role of Crosslinker Content of Positively Charged NIPAM Nanogels on the In Vivo Toxicity in Zebrafish

Roberta Bilardo [1], Federico Traldi [1], Caroline H. Brennan [2] and Marina Resmini [1,*]

[1] Department of Chemistry, School of Physical and Chemical Sciences, Queen Mary University of London, London E1 4NS, UK; r.bilardo@qmul.ac.uk (R.B.); f.traldi@qmul.ac.uk (F.T.)
[2] School of Biological and Behavioural Sciences, Queen Mary University of London, London E1 4NS, UK; c.h.brennan@qmul.ac.uk
* Correspondence: m.resmini@qmul.ac.uk

Table S1. Chemical composition, monomer conversions (^{1}H NMR) and chemical yields of positively charged NIPAM-based nanogels, containing 10 mol% *t*-BAEMA as functional monomer, before the optimization of the polymerization conditions. All nanogels were synthesized with MBA as the crosslinker in relative concentrations ranging between 5 – 20 mol%. For all the formulations, the initiator (AIBN) was 1% of the total moles of double bonds in the mixture, while the total monomer concentration (C_M) used was kept equal to 1 or 2%.

Nanogel	Composition (mol%)			C_M (%)	Monomer Conversion (%)				Yield (%)
	NIPAM	MBA	t-BAEMA		NIPAM	MBA	*t*-BAEMA	Overall	
NG_{20}-tBAEMA-1	70	20	10	1	67	88	97	75	64
NG_{10}-tBAEMA-1	80	10	10	1	60	75	98	65	48
NG_5-tBAEMA-1	85	5	10	1	55	68	97	59	29
NG_{20}-tBAEMA-2	70	20	10	2	79	95	99	84	79
NG_{10}-tBAEMA-2	80	10	10	2	74	91	99	79	62
NG_5-tBAEMA-2	85	5	10	2	70	83	99	74	50

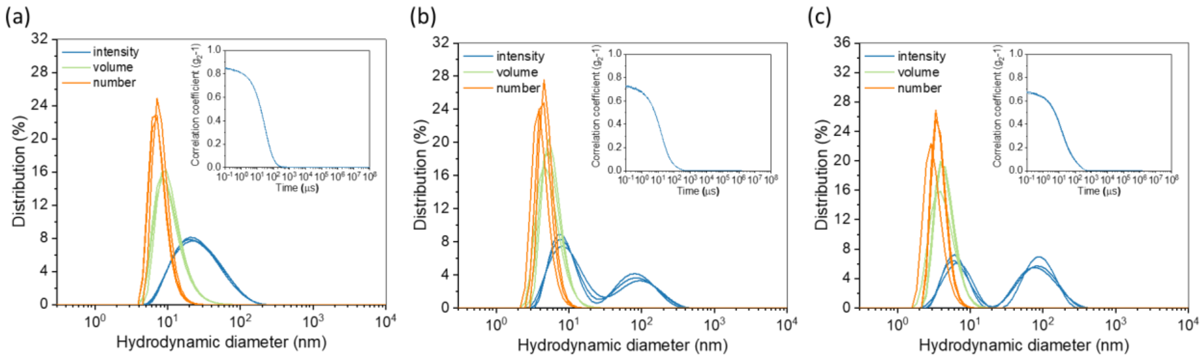

Figure S1. Triplicate DLS measurements of neutral nanogels, with crosslinker content between 5 and 20 mol%: **(a)** NG_{20}-1; **(b)** NG_{10}-1; and **(c)** NG_{5}-1 in deionized water (1 mg/mL, 25°C) by intensity (blue), volume (green) and number (orange) distributions. Inserts show correlogram of each triplicate measurement. The presence of a single peak by volume and number distributions suggests that the second population, observed by intensity for NG_{10}-1and NG_{5}-1, represents a negligible fraction of the samples.

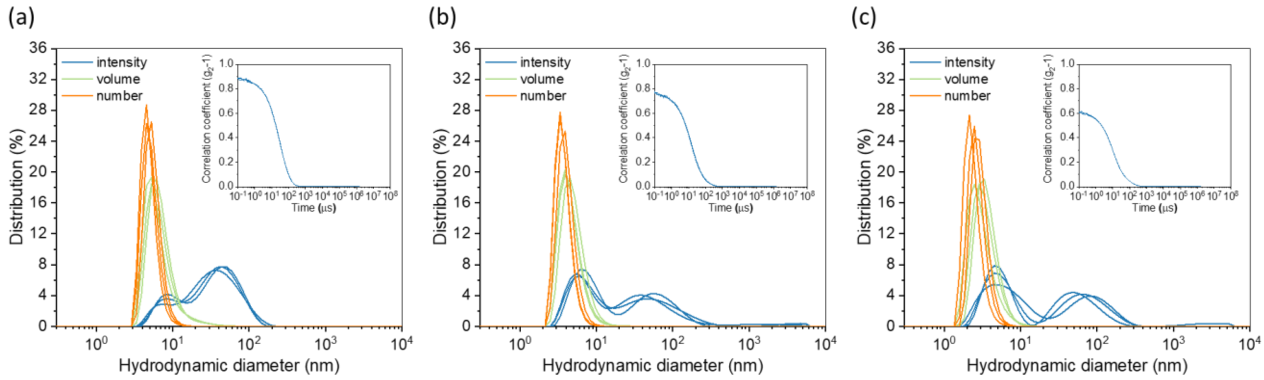

Figure S2. Triplicate DLS measurements of *t*-BAEMA nanogels, with crosslinker content between 5 and 20 mol%: **(a)** NG_{20}-tBAEMA-3; **(b)** NG_{10}- tBAEMA-3; and **(c)** NG_{5}- tBAEMA-3 in deionized water (1 mg/mL, 25°C) by intensity (blue), volume (green) and number (orange) distributions. Inserts show correlogram of each triplicate measurement. The presence of a single peak by volume and number distributions suggests that the second population, observed by intensity, represents a negligible fraction of the samples.

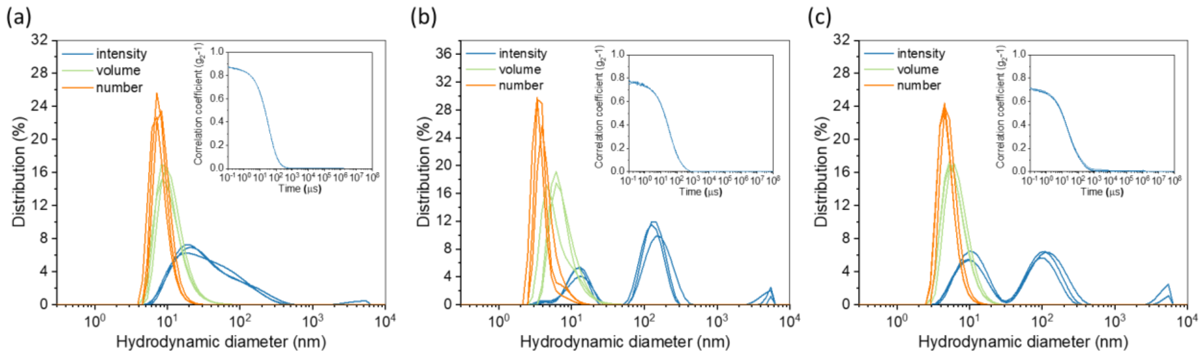

Figure S3. Triplicate DLS measurements of neutral nanogels, with crosslinker content between 5 and 20 mol%: **(a)** NG_{20}-1; **(b)** NG_{10}-1; and **(c)** NG_5-1 in fish water with pH = 7 (1 mg/mL, 25°C) by intensity (blue), volume (green) and number (orange) distributions. Inserts show correlogram of each triplicate measurement. The presence of a single peak by volume and number distributions suggests that the second population, observed by intensity, represents a negligible fraction of the samples.

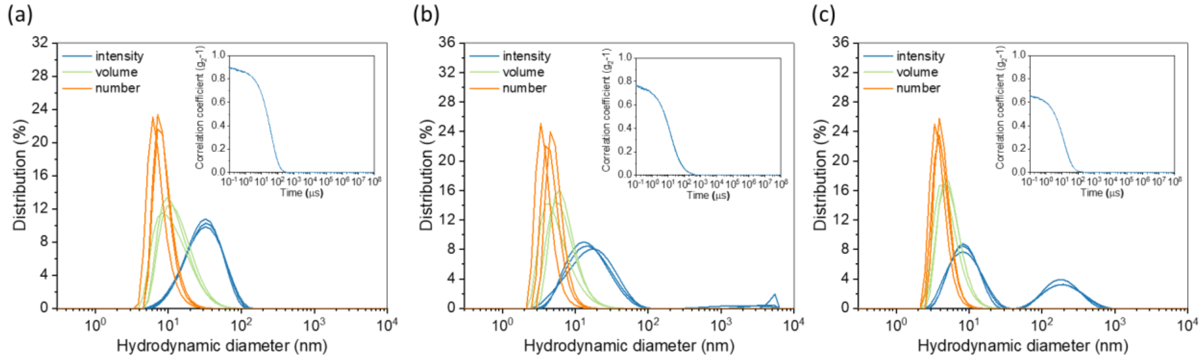

Figure S4. Triplicate DLS measurements of *t*-BAEMA nanogels, with crosslinker content between 5 and 20 mol%: **(a)** NG_{20}-tBAEMA-3; **(b)** NG_{10}- tBAEMA-3; and **(c)** NG_5- tBAEMA-3 in fish water with pH = 7 (1 mg/mL, 25°C) by intensity (blue), volume (green) and number (orange) distributions. Inserts show correlogram of each triplicate measurement. The presence of a single peak by volume and number distributions suggests that the second population, observed by intensity for NG_5-tBAEMA-3, represents a negligible fraction of the samples.

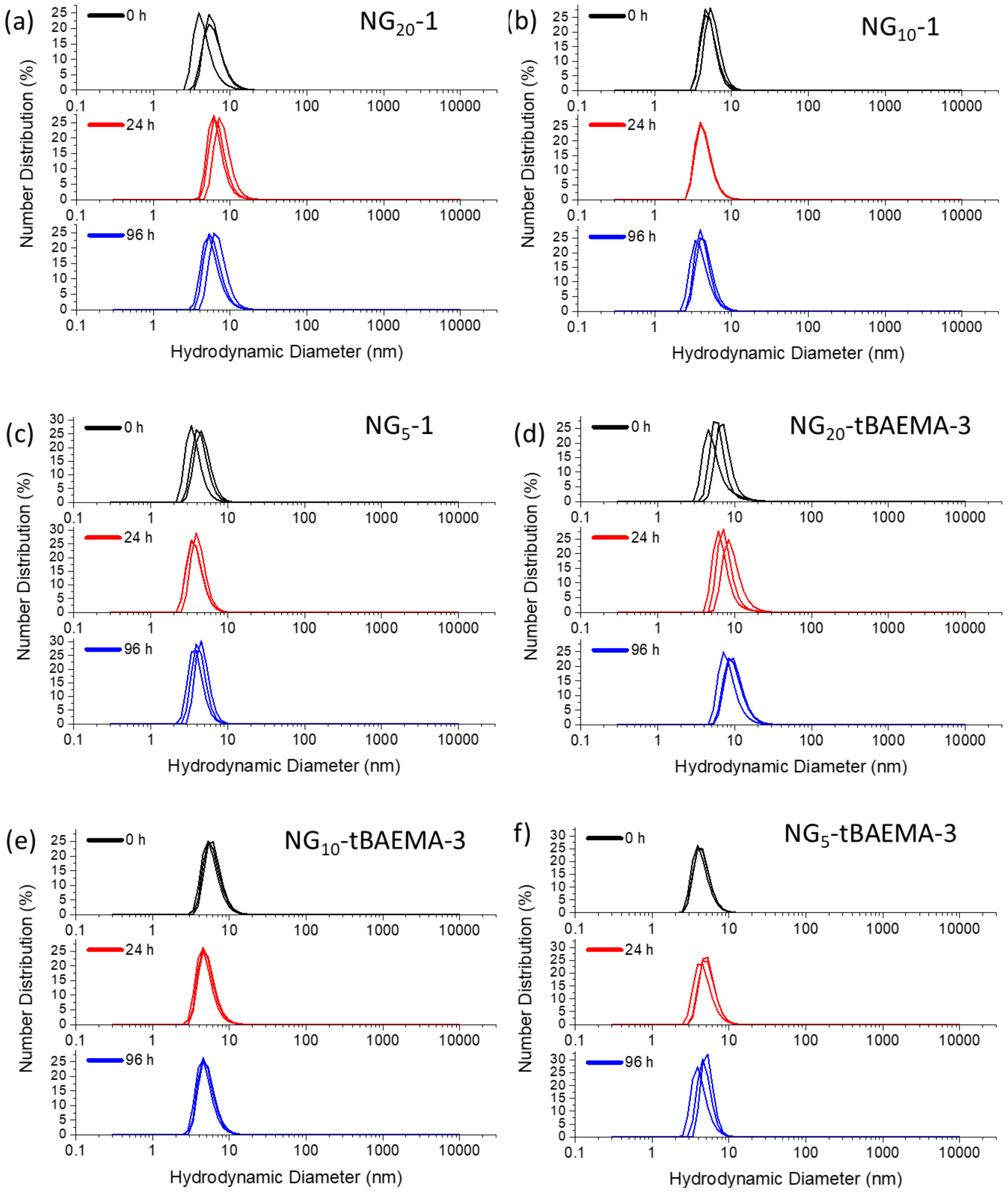
(a)
NG_{20}-1
(b)
NG_{10}-1
(c)
NG_{5}-1
(d)
NG_{20}-tBAEMA-3
(e)
NG_{10}-tBAEMA-3
f)
NG_{5}-tBAEMA-3
0 h
24 h
96 h
Number Distribution (%)
Hydrodynamic Diameter (nm)
0.1
1
10
100
1000
10000

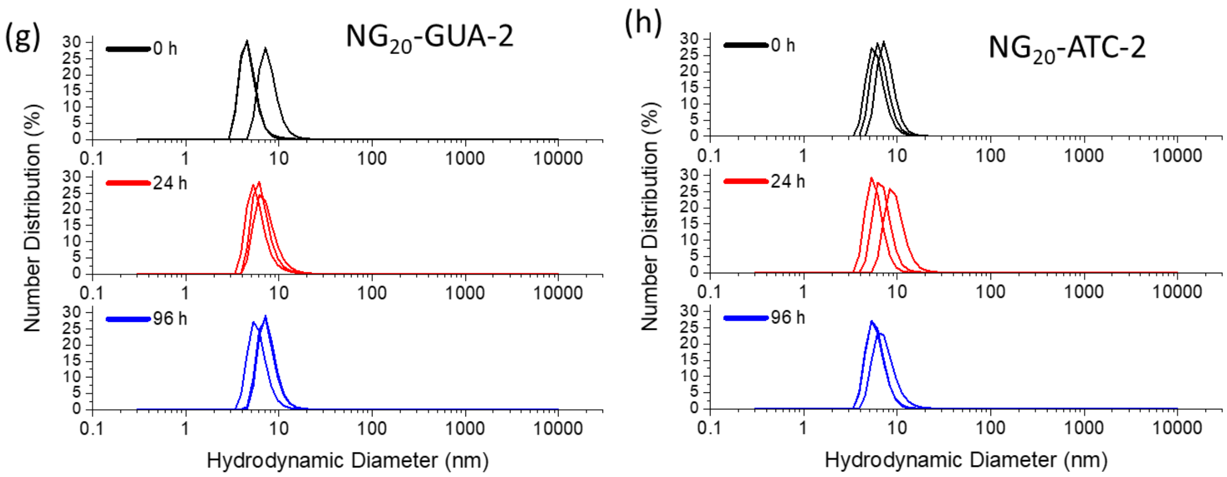

Figure S5. Colloidal stability studies of nanogels in fish water (pH = 7) at 28 °C (0.5 mg/mL). Triplicate DLS measurements (number distribution) of **(a)** NG_{20}-1; **(b)** NG_{10}-1; and **(c)** NG_{5}-1 **(d)** NG_{20}-tBAEMA-3; **(e)** NG_{10}- tBAEMA-3; and **(f)** NG_{5}- tBAEMA-3; **(g)** NG_{20}-GUA-2; **(h)** NG_{10}- ATC-2 were obtained after 0 (black), 24 (red), and 96 (blue) hours after reconstitution of the dry nanogel powders. Data show that nanogels are colloidal stable within the time frame of the *in vivo* toxicity studies.

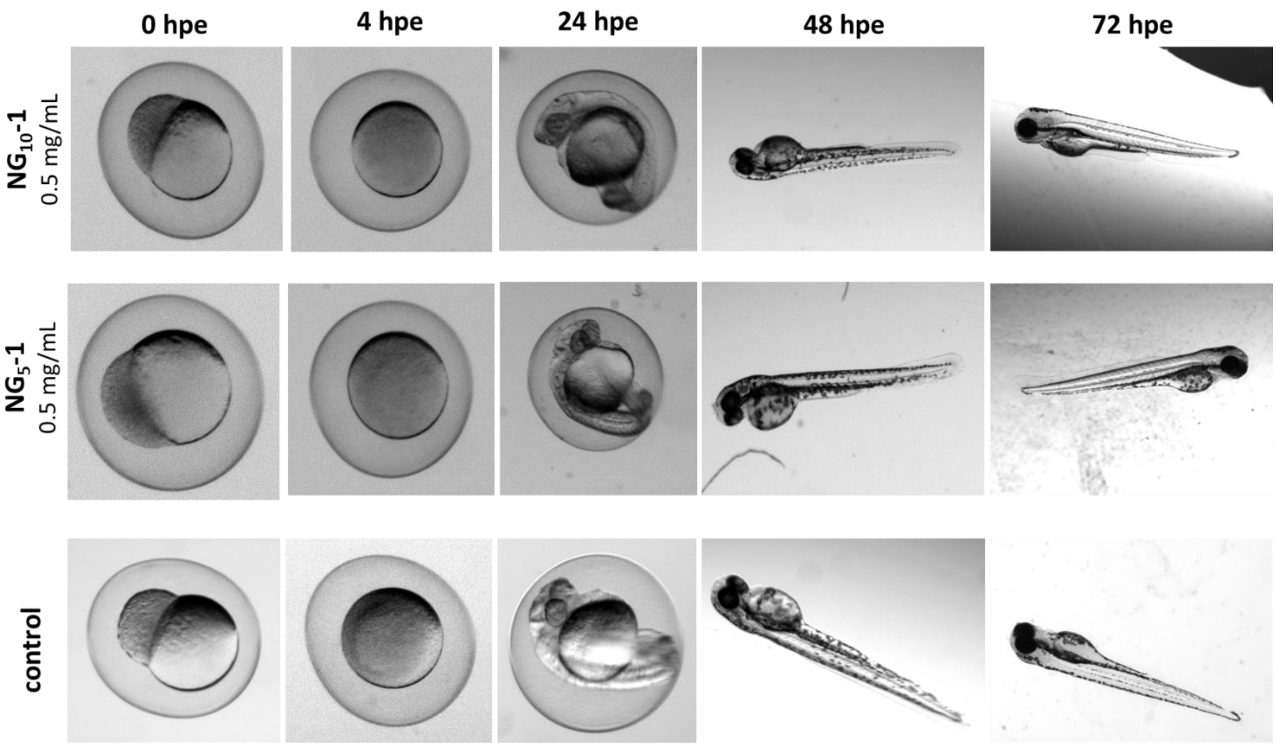

Figure S6. Stereomicroscopy assessment of the biocompatibility of neutral nanogels NG_{10}-1 (10 mol% crosslinker, first row) and NG_5-1 (5 mol% crosslinker, second row), in zebrafish up to 72 hours post exposure (hpe). The morphology and development of the embryos/larvae, exposed by immersion in nanogel solutions in fish water (concentration 0.5 mg/mL), was monitored at 0, 4 24, 48 and 72 hours after the administration and compared to non-exposed embryos/larvae used as negative control. These data are representative of the biocompatibility of neutral nanogels also at lower concentrations, *i.e.* 0.3 mg/mL and 0.1 mg/mL.

Table S2. Chemical composition, monomer conversions (^{1}H NMR) and chemical yields of positively charged NIPAM-based nanogels, containing 10 mol% of either *t*-BAEMA, or GUA, or ATC as functional monomer. All nanogels were synthesized keeping the crosslinker (MBA) content equal to 20 mol%. For each formulation, the optimization of the polymerizations led to a concentration of the initiator (AIBN) and a total monomer concentration (C_M) between 1 and 2 mol% (of the total moles of double bonds in the mixture) and between 2 and 3%, respectively. The individual monomer conversion of ATC could not be determined because of the overlapping of its peaks with the ones of MBA.

Nanogel	Composition (mol%)			AIBN (mol%)	C_M (%)	Monomer Conversion (%)				Yield (%)
	NIPAM	MBA	Co-monomer			NIPAM	MBA	Co-monomer	Overall	
			t-BAEMA					t-BAEMA		
NG_{20}-tBAEMA-3	70	20	10	1	3	90	98	99	92	77
			GUA					GUA		
NG_{20}-GUA-2	70	20	10	2	2	89	97	94	91	60
			ATC					ATC		
NG_{20}-ATC-2	70	20	10	2	2	86	97	-	89	54

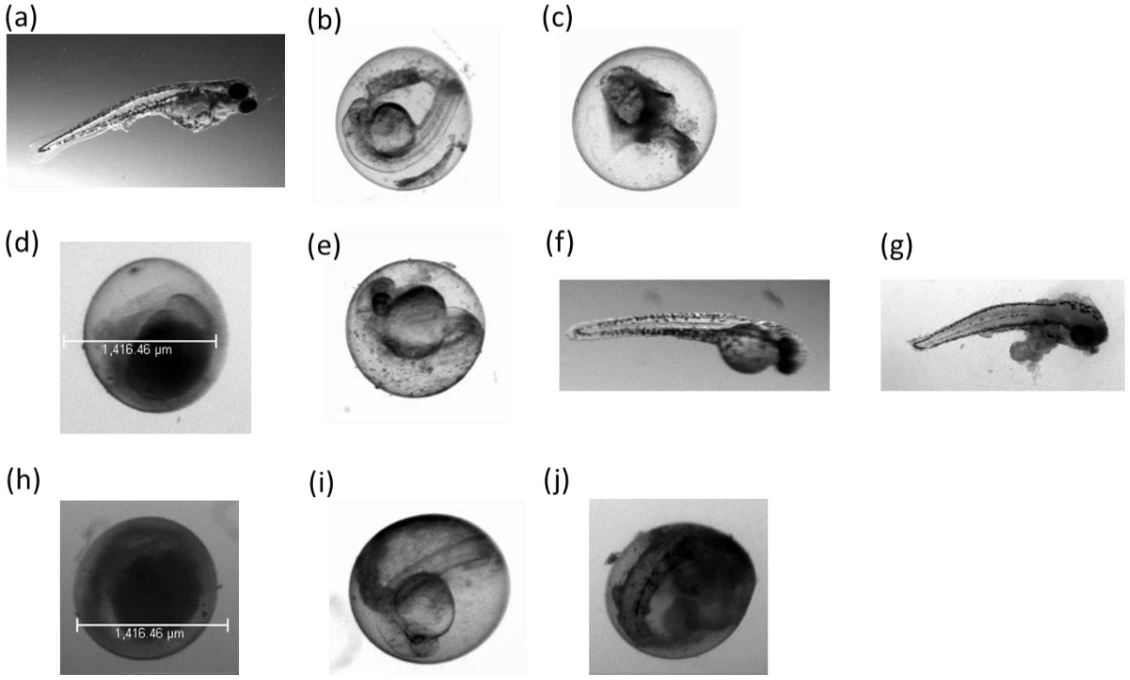

Figure S7. Examples of embryos/larvae dead after the immersion into nanogels solutions, for different times. In particular, the reported pictures show embryos/larvae exposed to : **(a)** NG_{20}-tBAEMA-3 0.1 mg/mL for 96 h; **(b)** NG_{10}-tBAEMA-3 0.1 mg/mL for 24 h; **(c)** NG_{5}-tBAEMA-3 0.1 mg/mL for 24 h; **(d)** NG_{20}-ATC-2 0.1 mg/mL for 4 h; **(e)** NG_{20}-ATC-2 0.1 mg/mL for 24 h; **(f)** NG_{20}-ATC-2 0.05 mg/mL for 48 h; **(g)** NG_{20}-ATC-2 0.03 mg/mL for 72 h; **(h)** NG_{20}-GUA-2 0.1 mg/mL for 4 h; **(i)** NG_{20}-GUA-2 0.1 mg/mL for 24 h; **(j)** NG_{20}-GUA-2 0.05 mg/mL for 48 h. Panels **(d)**, **(g)**, **(h)** and **(j)** were found to be necrotic, while the other panels show embryos/larvae with normal morphology but appearing to be disintegrating, as common after the death.

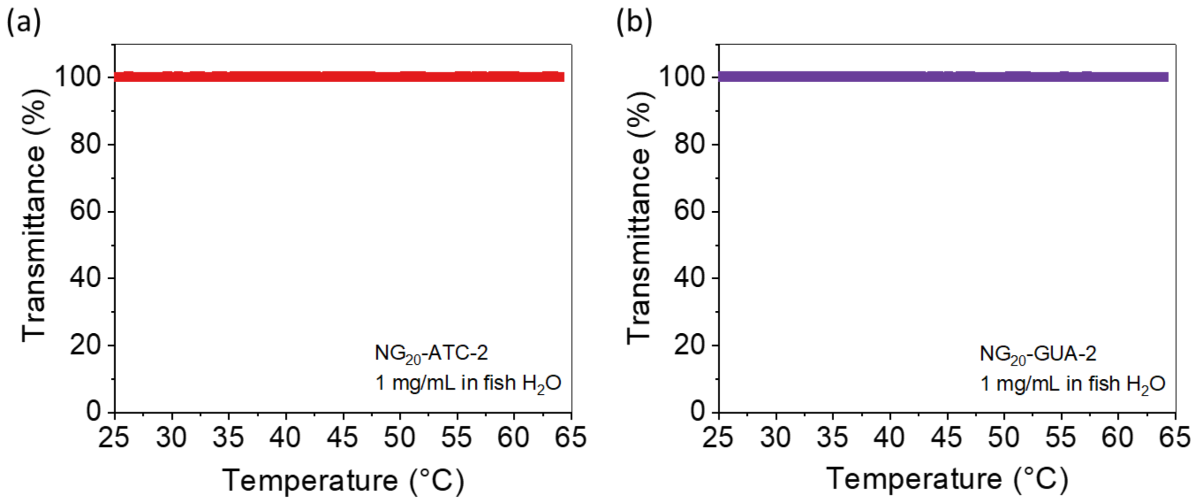

Figure S8. UV-visible spectroscopy study of the thermo responsive properties of the positively charged nanogels, containing 20 mol% of crosslinker, **(a)** NG_{20}-ATC-2 and **(b)** NG_{20}-GUA-2 in fish water (concentration 1 mg/mL), in a temperature range between 25 and 65°C.

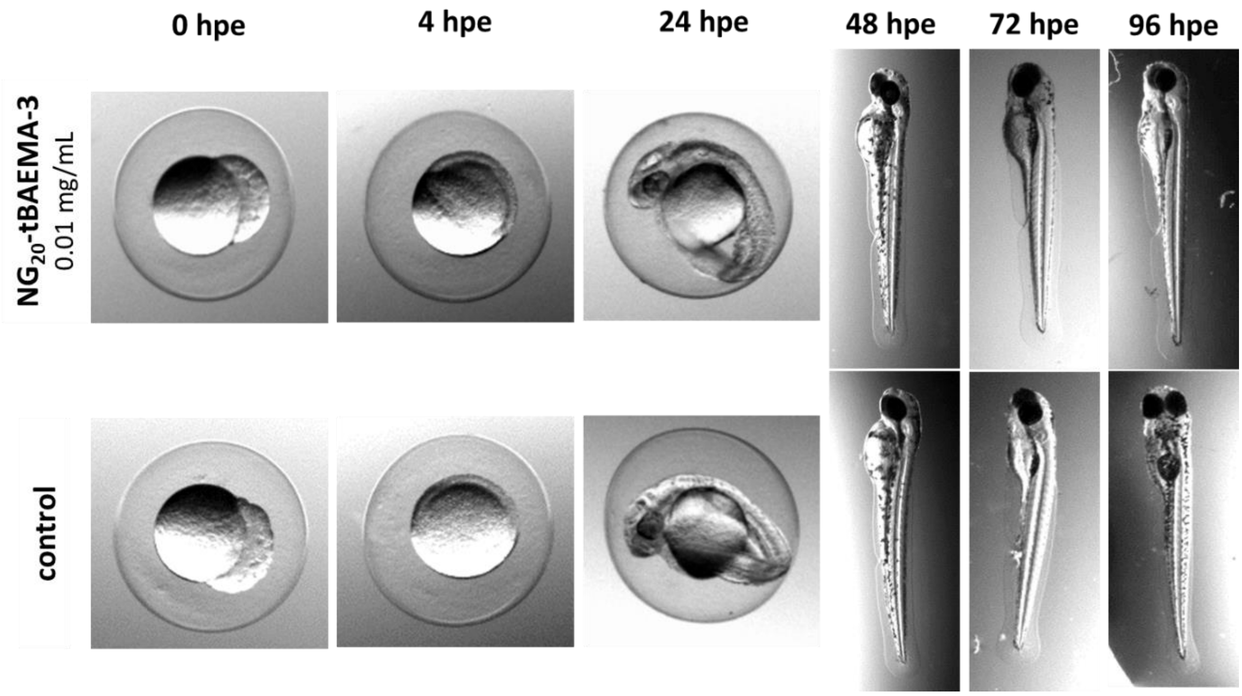

Figure S9. Stereomicroscopy assessment of the biocompatibility of the positively charged nanogel NG_{20}-tBAEMA-3 (20 mol% crosslinker, and 10 mol% of *t*-BAEMA), in zebrafish up to 96 hours post exposure (hpe). The morphology and development of the embryos/larvae, exposed by immersion in nanogel solutions in fish water (concentration 0.01 mg/mL), was monitored at 0, 4 24, 48, 72 and 96 hours after the administration and compared to non-exposed embryos/larvae used as negative control.
